# Burden and Distribution of Protozoan Pathogens in Diarrhea Cases Worldwide: A Systematic Review and Meta-Analysis, 1999-2024

**DOI:** 10.7759/cureus.91561

**Published:** 2025-09-03

**Authors:** Joseph B Suleiman, Maryam Azlan

**Affiliations:** 1 School of Health Sciences, Universiti Sains Malaysia, Kelantan, MYS; 2 Department of Science Laboratory Technology, Akanu Ibiam Federal Polytechnic, Unwana, Ebonyi, NGA

**Keywords:** diarrhea, meta analysis, pathogens, protozoa, systematic review

## Abstract

Protozoan pathogens are significant contributors to global diarrheal morbidity and mortality, particularly in resource-limited settings. Despite their clinical importance, the global burden and geographic distribution of protozoan-related diarrhea remain incompletely characterized. This study aimed to quantify the prevalence and regional trends of key protozoan pathogens in diarrheal cases globally from 1999 to 2024. A comprehensive systematic review and meta-analysis were conducted following PRISMA guidelines. Five databases (PubMed, Scopus, Google Scholar, Web of Science, and ScienceDirect) were searched for studies reporting the prevalence of *Giardia duodenalis, Entamoeba histolytica, Cryptosporidium* spp., *Blastocystis hominis,* and *Cyclospora cayetanensis* in patients with diarrhea. Random-effects models were used to estimate pooled prevalence, with subgroup analyses by region, age, diagnostic method, and socioeconomic indicators. Heterogeneity and publication bias were assessed using meta-regression and funnel plots. Results of the meta-analysis of 73 studies revealed a global protozoan prevalence of 7.5% (95% CI: 5.6%-10.0%) in diarrheal cases, with the highest rates in the Americas and Africa. *Giardia* and *Cryptosporidium* were the most common pathogens. Despite substantial heterogeneity and some small-study bias, findings were robust, with minimal publication bias and variation due to diagnostic methods used. Protozoan pathogens remain major yet underrecognized drivers of diarrheal disease worldwide. Targeted interventions, including improved diagnostics, sanitation, and surveillance, are essential to mitigate their impact.

## Introduction and background

Diarrheal diseases caused by protozoan pathogens represent a persistent global health challenge, particularly in resource-limited settings where poor sanitation and inadequate water infrastructure facilitate transmission [1-3]. Among the most clinically significant enteric protozoa, *Cryptosporidium* spp., *Giardia duodenalis,* and *Entamoeba histolytica* collectively account for an estimated 500 million annual diarrheal cases worldwide, contributing substantially to childhood morbidity, malnutrition, and developmental delays [4-6]. These pathogens disproportionately affect children under five in low- and middle-income countries (LMICs), where they are responsible for 10-15% of diarrheal deaths and are increasingly recognized as contributors to long-term growth faltering and cognitive impairment [7,8]. Despite their significant disease burden, protozoan enteropathogens remain understudied compared to bacterial and viral agents, with critical gaps in our understanding of their spatiotemporal distribution, zoonotic transmission dynamics, and interactions with environmental and socioeconomic factors [8].

The epidemiology of protozoan enteric infections reveals striking geographical disparities. *Cryptosporidium* alone causes approximately 200,000 deaths annually, with the highest burden in sub-Saharan Africa and South Asia [9,10]. Recent studies demonstrate that cryptosporidiosis is associated with a 2-3 times higher risk of mortality in malnourished children compared to other diarrheal etiologies [11-13]. *Giardia* infections, while less frequently fatal, affect an estimated 280 million people each year and are linked to chronic malnutrition, micronutrient deficiencies, and post-infectious irritable bowel syndrome [14,15]. *Entamoeba histolytica*, though geographically more restricted, remains a significant cause of dysentery and extra-intestinal complications, particularly in Central and South America and parts of Asia [15]. The true burden of these pathogens is likely underestimated due to diagnostic challenges, with microscopy-based surveillance missing 30-50% of cases detectable by molecular methods [16].

Protozoan enteropathogens exhibit complex transmission patterns influenced by environmental, climatic, and anthropogenic factors. *Cryptosporidium* oocysts and *Giardia* cysts are remarkably resistant to standard water treatment methods, leading to frequent waterborne outbreaks even in high-income countries [17]. Climate change is altering transmission dynamics, with studies linking increased rainfall intensity to *Cryptosporidium* outbreaks and drought conditions to *Giardia* proliferation [18]. Zoonotic transmission plays a significant role, particularly for *Cryptosporidium parvum* and *Giardia* assemblages with animal reservoirs, creating challenges for One Health approaches to disease control [19]. Similarly, urbanization has introduced new transmission patterns, with dense informal settlements creating ideal conditions for person-to-person spread of *Entamoeba histolytica* [20]. Some enteric protozoans, their global prevalence, and associated health risks are summarized in Table 1.

**Table 1:** Global prevalence and health risks of enteric protozoa and related organisms.

Enteric organisms	Global prevalence	Effects on humans	Risk level
Cyclospora cayetanensis	Rare (<1%); outbreaks in Latin America, Asia, USA	Causes prolonged watery diarrhea, abdominal cramps, fatigue	Pathogenic
Cystoisospora belli (formerly Isospora belli)	Very rare (<0.5%); mostly in tropics	Severe diarrhea in immunocompromised individuals (e.g., HIV/AIDS)	Opportunistic pathogen
Giardia duodenalis (syn. G. lamblia)	Common: 2-7% in developed, 30-40% in developing countries	*Giardiasis* - watery diarrhea, bloating, malabsorption	Pathogenic
Blastocystis spp. (formerly B. hominis)	Very common: 10-60% worldwide	Sometimes causes diarrhea and abdominal pain; often asymptomatic	Possibly pathogenic
Entamoeba histolytica	About 1-2% true infections (10% carry Entamoeba species)	Amoebiasis - bloody diarrhea, dysentery, liver abscess	Pathogenic
Cryptosporidium parvum	1-4% worldwide; up to 10% in children in low-income regions	Severe watery diarrhea; life-threatening in immunocompromised patients	Pathogenic
Endolimax nana	Very common: up to 30-40%	Harmless; indicator of poor hygiene/fecal exposure	Nonpathogenic
Entamoeba hartmanni	Common: 5-10%	Harmless; resembles *E. histolytica* microscopically	Nonpathogenic
Chilomastix mesnili	Found in ~5%	Harmless intestinal commensal	Nonpathogenic
Pentatrichomonas hominis (formerly Trichomonas hominis)	Fairly common: 1-10%	Harmless intestinal commensal	Nonpathogenic

Furthermore, the advent of molecular diagnostics has revolutionized protozoan detection, revealing higher prevalence rates and more frequent polyparasitism than previously recognized. Multiplex PCR studies demonstrate that 15-25% of diarrheal cases in endemic areas involve protozoan co-infections, often alongside bacterial or viral pathogens [20,21]. Despite these diagnostic advances, treatment options remain limited, nitazoxanide is the only FDA-approved drug for cryptosporidiosis, and resistance is emerging [22]. The lack of vaccines for any protozoan enteropathogen underscores the critical need for preventive strategies targeting water, sanitation, and hygiene (WASH) interventions [23]. Recent trials of monoclonal antibodies for cryptosporidiosis prevention show promise but face implementation challenges in high-burden settings [24,25].

## Review

Methodology

Search Strategy and Data Sources

A systematic literature search was conducted across five major electronic databases: Google Scholar, PubMed, Scopus, Web of Science, and ScienceDirect. The search strategy was structured around three primary concept clusters: (1) terms related to co-infection (e.g., “Coinfection,” “Concurrent infection,” “Dual infection,” “Mixed infection”), (2) specific pathogens (e.g., Vibrio cholerae, Shigella spp., Enteropathogenic E. coli, Rotavirus, Norovirus, Cryptosporidium, Giardia), and (3) epidemiological measures (e.g., “Prevalence,” “Incidence,” “Burden,” “Epidemiology”). No restrictions were imposed regarding language, publication date, or study design. The final search update, completed on December 30, 2024, identified 1,133 potentially eligible articles.

Study Design and Registration

This systematic review and meta-analysis adhered to the Preferred Reporting Items for Systematic Reviews and Meta-Analyses (PRISMA) guidelines. The protocol was prospectively registered with PROSPERO (registration ID: CRD420251060392) to enhance methodological transparency and minimize research duplication. The review aimed to assess the global prevalence of protozoan enteric pathogens in diarrheal cases reported from January 1999 through December 2024.

Study Selection and Eligibility Criteria

Inclusion criteria: Eligible studies met the following criteria: (1) original research articles reporting laboratory-confirmed detection of enteric pathogens, (2) inclusion of at least two identified pathogens per diarrheal case, and (3) population-based studies with clearly defined diagnostic methods and data collected between 1999 and 2024.

Exclusion criteria:* *The following were excluded: reviews, editorials, case reports, and other non-primary research; studies focused solely on animals or environmental samples; articles with incomplete prevalence data or ambiguous diagnostic methods; and duplicate publications based on overlapping datasets.

Screening Process 

Two independent reviewers screened titles and abstracts, followed by full-text assessments of potentially relevant articles. Discrepancies were resolved through consensus discussions within the research team. The selection process was documented using a PRISMA flow diagram.

Data extraction and management 

Data were extracted using a standardized form capturing study characteristics (e.g., author, year, country, study design), population details (e.g., sample size, age distribution, clinical features), and pathogen information (e.g., detection methods, co-infection rates, sampling strategies, laboratory protocols). Two researchers independently verified the extracted data to ensure consistency and accuracy.

Quality assessment 

The methodological quality of included studies was assessed using the Joanna Briggs Institute (JBI) Critical Appraisal Checklist for Prevalence Studies, covering nine key domains. Studies scoring ≥7 out of 9 were deemed high quality and included in the quantitative synthesis. Quality assessments were conducted independently by two reviewers, achieving strong inter-rater agreement (Cohen’s κ = 0.82). Further details are provided in Appendix 1.

Statistical analysis 

Meta-analysis Approach 

Random-effects meta-analyses using the DerSimonian-Laird method were performed to account for heterogeneity between studies. Pooled prevalence estimates were calculated using inverse-variance weighting. Subgroup analyses were conducted based on pathogen type, geographical region, and time period.

Heterogeneity and Publication Bias 

Statistical heterogeneity was quantified using the I² statistic, with thresholds of 25%, 50%, and 75% representing low, moderate, and high heterogeneity, respectively. Publication bias was assessed via funnel plot asymmetry and Egger’s regression test. Sensitivity analyses were conducted by sequentially excluding individual studies to assess the robustness of the results.

Software Tools 

Primary meta-analyses were conducted using OpenMeta[Analyst]. Jamovi version 2.3.28 was used for publication bias assessments, while meta-regression analyses were performed using OpenMEE software. In addition, MapChart software was used to design and visualize the global distribution maps of protozoan enteropathogens.

Ethical considerations 

This study relied solely on previously published, de-identified data and therefore did not require formal ethical approval. All sources were appropriately cited in accordance with copyright and academic integrity standards.

Results

Study Selection Process

**Figure 1:**
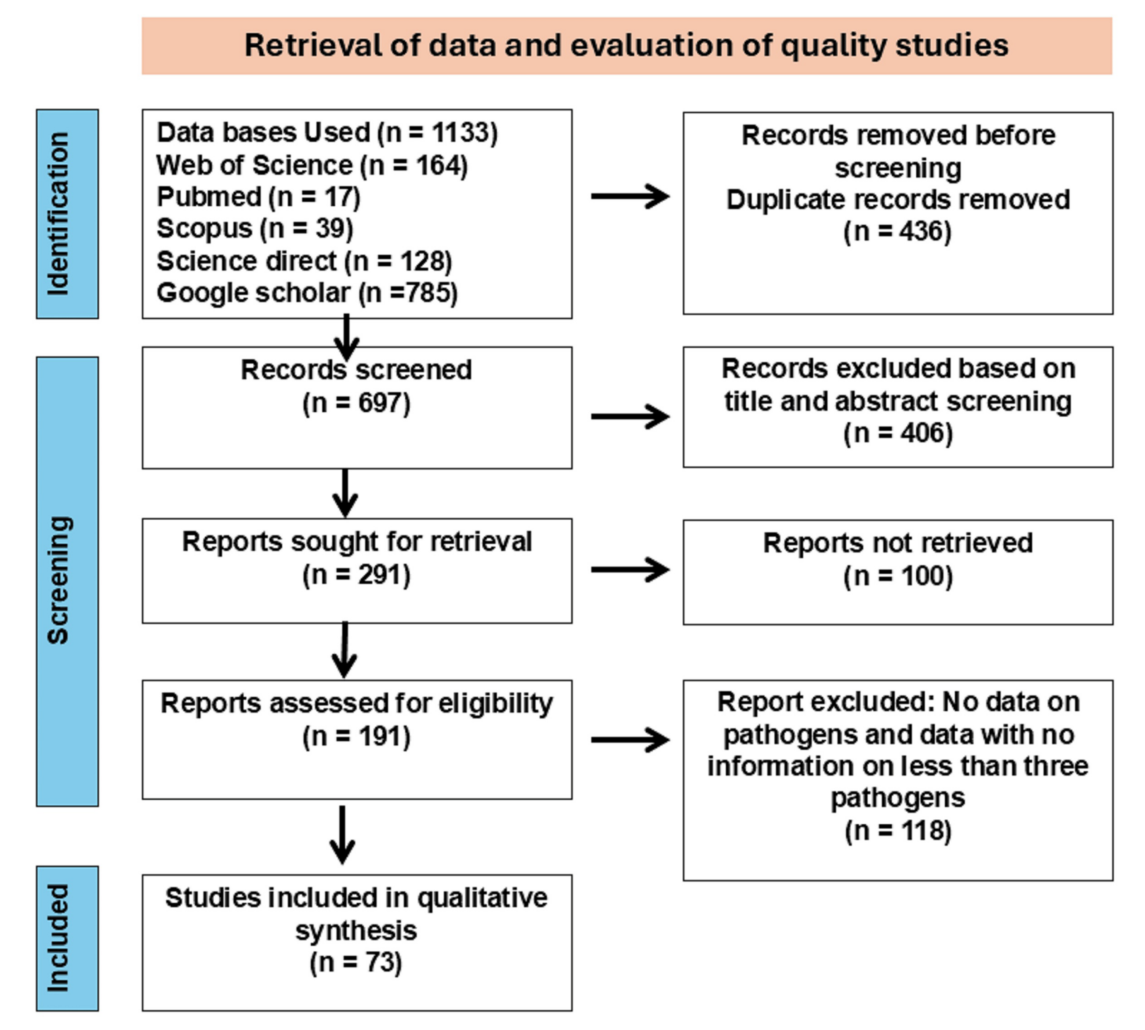
Summary of the procedure for identifying and selecting relevant articles.

Figure 1 presents the PRISMA flow diagram detailing the systematic identification and screening of articles. Our comprehensive search across five electronic databases initially yielded 1,133 records. After duplicate removal and application of inclusion and exclusion criteria, 697 full-text articles underwent rigorous eligibility assessment. Ultimately, 73 studies met all criteria for inclusion in both the qualitative synthesis and meta-analysis (Appendix 2).

Characteristics of Included Studies

The incorporated studies exhibited the following key characteristics: (1) studies covered nearly all regions of the world, i.e., the Americas, Asia, Europe, and Africa; (2) all studies were conducted between 1999 and 2024; (3) clinical samples consisted of stool specimens from humans. Furthermore, the primary focus was on bacterial pathogens. Detection methods such as conventional disk diffusion, PCR, and ELISA are summarized in Table 2 and Appendix 3.

**Table 2:** Distribution of global protozoan isolates from 1999-2024. Source: References [26-85]

Author	Publication Year	Country	Continent	Detection methods	Sample size	Pathogen isolates	Protozoan isolates
Shrestha et al. A [26]	2022	Nepal	Asia	Culture	1,200	1,254	119
Shrestha et al. B [26]	2022	Nepal	Asia	ELISA/PCR	1,200	799	211
Farfán-García et al. A [27]	2020	Colombia	America	ELISA/PCR	431	547	117
Farfán-García et al. B [27]	2020	Colombia	America	Culture	430	346	111
Albert et al. A [28]	1999	Bangladesh	Asia	Culture	814	992	23
Albert et al. B [28]	1999	Bangladesh	Asia	ELISA/Culture	814	479	27
Verma et al. [29]	2019	India (North)	Asia	Culture/PCR	100	73	5
Huhulescu et al. [30]	2009	Austria	Europe	PCR	306	75	4
Aktaş et al. [31]	2019	Turkey	Europe	ELISA/PCR	375	265	2
Tam et al. A [32]	2012	England	Europe	Culture/PCR/Microscopy	874	784	21
Tam et al. B [32]	2012	England	Europe	PCR	782	479	23
Hawash et al. [33]	2017	Saudi Arabia	Asia	ELISA/PCR	163	107	45
Youssef et al. [34]	2000	Jordan	Asia	ELISA/PCR	265	217	19
Williams et al. [35]	2020	Democratic Republic of the Congo	Africa	Culture/PCR/Microscopy	269	391	74
Chopra et al. [36]	2013	India	Asia	ELISA/PCR	200	119	55
Ng’ang’a et al. [37]	2018	Kenya	Africa	Culture/PCR/Microscopy	174	207	1
Ajjampur et al. [38]	2009	India	Asia	PCR	452	114	17
Al-Gallas et al. A [39]	2007	Tunisia	Africa	PCR	271	217	2
Al-Gallas et al. B [39]	2007	Tunisia	Africa	PCR	271	108	2
Torres et al. [40]	2001	Uruguay	America	Culture/PCR	224	215	27
Potgieter et al. [41]	2023	South Africa	Africa	Culture/PCR	275	56	2
Makhari et al. [42]	2012	South Africa	Africa	PCR	2,468	1,081	85
Shrivastava et al. [43]	2017	India	Asia	Culture	130	77	5
Huang et al. A [44]	2018	Taiwan	Asia	PCR	217	87	1
Huang et al. B [44]	2018	Taiwan	Asia	PCR	217	121	1
Zboromyrska et al. [45]	2014	Spain	Europe	ELISA/PCR	185	76	18
Abraham et al. [46]	2024	India	Asia	PCR	2,694	1,812	90
Lanata et al. [47]	2025	Peru	America	PCR	676	945	68
Piralla et al. [48]	2017	Italy	Europe	PCR	168	108	9
Pelkonen et al. A [49]	2018	Angola	Africa	PCR	98	284	31
Pelkonen et al. B [49]	2018	Angola	Africa	PCR	96	166	18
Iqbal et al. [50]	2024	Pakistan	Asia	PCR	245	1,089	137
Alsuwaidi et al. A [51]	2021	UAE	Asia	PCR	203	279	31
Alsuwaidi et al. B [51]	2021	UAE	Asia	ELISA/PCR	73	35	1
Abbasi et al. [52]	2022	Iran	Asia	PCR	211	257	15
Casillas-Verga et al. [53]	2020	Mexico	America	Culture	240	103	12
Jennings et al. [54]	2017	Peru	America	PCR	230	148	16
Eibach et al. A [55]	2016	Ghana	Africa	PCR	443	1,106	262
Eibach et al. B [55]	2016	Ghana	Africa	PCR	239	621	205
Kara et al. [56]	2022	Turkey	Europe	PCR	203	203	7
Khare et al. A [57]	2014	USA	America	PCR	230	91	3
Khare et al. B [57]	2014	USA	America	PCR	230	77	3
Tilmanne et al. A [58]	2019	Belgium	Europe	Culture	178	126	17
Tilmanne et al. B [58]	2019	Belgium	Europe	Culture/PCR	178	78	4
Castany-Feixas et al. [59]	2021	Spain	Europe	PCR	125	88	13
Knee et al. [60]	2018	Mozambique	Africa	PCR	759	183	55
Sameer et al. [61]	2024	Bahrain	Asia	Culture/PCR	109	143	3
Japa et al. [62]	2024	Dominican Republic	America	Culture/PCR	170	227	21
Benmessaoud et al. [63]	2015	Morocco	Africa	PCR	122	123	2
Leli et al. [64]	2020	Italy	Europe	PCR	183	82	2
Buuck et al. [65]	2020	USA	America	PCR	224	242	2
Pativada et al. [66]	2012	India	Asia	PCR	2,535	51	8
Toffel et al. [67]	2019	USA	America	Culture/PCR	222	199	14
Nair et al. [68]	2010	India	Asia	PCR	2,536	2,829	532
Santos et al. [69]	2019	Brazil	America	PCR	591	607	82
Tsagarakis et al. [70]	2017	Greece	Europe	PCR	1,041	2,295	37
Meyer et al. [71]	2022	Switzerland	Europe	PCR	179	134	2
Carmon et al. [72]	2023	Israel	Asia	PCR	91	89	1
Mladenova et al. [73]	2015	Bulgaria	Europe	PCR	92	67	3
Fidalgo et al. [74]	2021	Spain	Europe	Culture/PCR	10,659	1,001	515
McAuliffe et al. [75]	2013	New Zealand	Europe	Culture	1,758	890	130
Holland et al. [76]	2020	United Kingdom	Europe	PCR	566,000	27,879	166
Zizza et al. [77]	2024	Italy	Europe	PCR	103	81	8
Olesen et al. [78]	2005	Denmark	Europe	PCR	424	220	8
Pouletty et al. [79]	2019	France	Europe	PCR	59	181	19
Mihala et al. [80]	2022	Australia	Asia	PCR	154	2,795	57
Nejma et al. A [81]	2014	Tunisia	Africa	PCR	124	296	4
Nejma et al. B [81]	2014	Tunisia	Africa	Culture	54	75	3
Saeed et al. [82]	2019	Libya	Africa	PCR	505	46	30
Brurce et al. A [83]	2016	Central African Republic	Africa	PCR	333	385	50
Brurce et al. B [83]	2016	Central African Republic	Africa	PCR	333	183	41
Murphy et al. [84]	2017	USA	America	PCR	2,257	1,127	48
Albert et al. A [85]	2016	Kuwait	Asia	Culture	109	20	3

Forest Plot of Global Protozoan Prevalence

**Figure 2:**
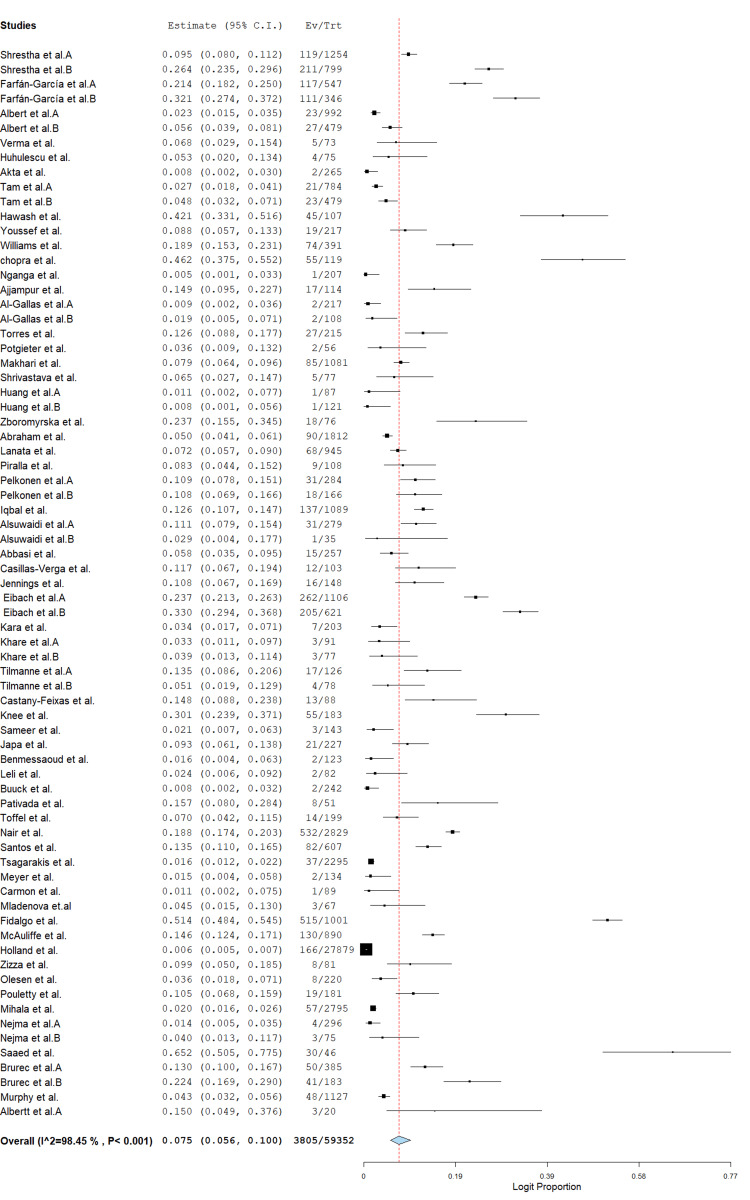
Overall prevalence of global protozoan isolates in diarrhea cases. The forest plot was generated using OpenMeta[Analyst] software. Source: References [26-85].

**Figure 3:**
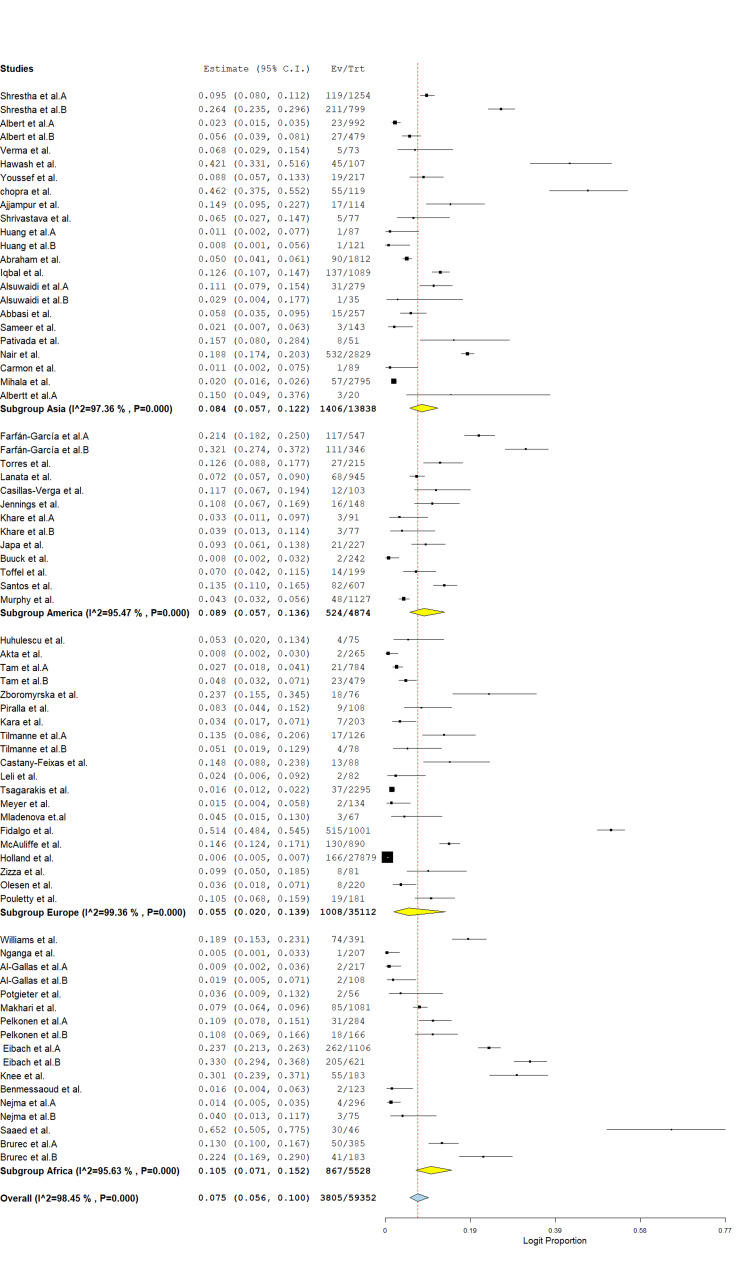
Subgroup prevalence of global protozoan isolates in diarrhea cases. The forest plot was generated using OpenMeta[Analyst] software. Source: References [26-85].

A total of 73 studies involving 59,352 stool samples from patients with diarrhea were included in the meta-analysis. The overall pooled prevalence of protozoa was 7.5% (95% CI: 5.6%-10.0%; logit proportion = 0.075), with significant heterogeneity across studies (I² = 98.4%, p < 0.001) (Figure 2). Subgroup analysis by region showed the highest prevalence in the Americas (12.0%, 95% CI: 6.4%-21.5%; I² = 95.4%), followed by Africa (10.6%, 95% CI: 6.7%-16.5%; I² = 95.3%), Asia (5.6%, 95% CI: 3.6%-8.5%; I² = 97.4%), and Europe (5.6%, 95% CI: 4.1%-7.6%; I² = 96.3%) (Figure 3). The high between-study heterogeneity likely reflects variations in geographic settings, study periods, diagnostic methods, and study populations. Despite this heterogeneity, these findings underscore protozoa as significant contributors to diarrheal disease globally, particularly in the Americas and Africa.

Funnel plot

**Figure 4:**
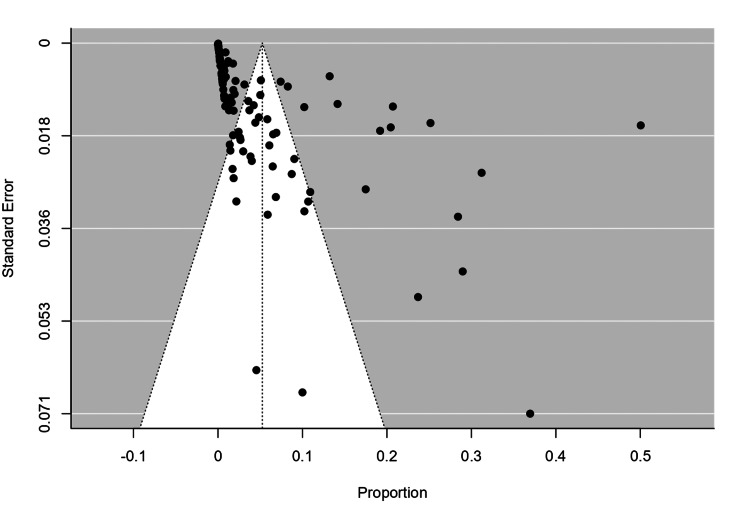
Funnel plot for global protozoan isolates in diarrhea cases. The funnel plot was generated using Jamovi version 2.3.28 software.

The funnel plot analysis revealed that the fail-safe N was extremely high (5,078,779, p < 0.001), indicating strong robustness of the meta-analysis findings against unpublished null studies. The rank correlation test (Kendall’s Tau = −0.249, p < 0.001) suggested possible funnel plot asymmetry and potential publication bias. However, Egger’s regression test showed no significant asymmetry (Z = 0.131, p = 0.896), indicating no evidence of bias. Despite some indication of asymmetry, the high fail-safe N supports the conclusion that the results are stable and unlikely to be substantially affected by missing or unpublished studies (Figure 4).

Subgroup Prevalence of Protozoa in Asia

This meta-analysis evaluated the prevalence of key protozoan pathogens among diarrheal cases in Asia. The most studied pathogen was *Cryptosporidium parvum*, reported in 19 studies with a pooled prevalence of 46.2% (95% CI: 33.6%-59.3%; I² = 89.74%, p < 0.001), indicating high heterogeneity. *Giardia lamblia* and *Giardia duodenalis* were reported in 14 and 13 studies, with prevalences of 52.1% (95% CI: 36.3%-67.9%; I² = 97.08%) and 53.4% (95% CI: 37.6%-68.6%; I² = 91.25%), respectively. *Entamoeba histolytica* had a lower pooled prevalence of 13.9% (95% CI: 6.3%-27.8%) across 10 studies (I² = 83.08%). Less frequently reported pathogens included *Cyclospora cayetanensis* (5.0%; 95% CI: 2.4%-10.2%; I² = 54.07%) and *Blastocystis hominis* (10.5%; 95% CI: 1.8%-42.8%; I² = 95.5%). *Isospora* was identified in only one study with a prevalence of 21.8% (95% CI: 12.8%-34.6%). Several protozoa were not reported. High heterogeneity suggests geographic and methodological variability across studies (Table 3).

**Table 3:** Pooled data on the prevalence of protozoan isolates in Asia.

Name of enteric pathogen	No. of studies	Prevalence (%)	95% CI	Heterogeneity test (I²)	p-value
Cyclospora cayetanensis	3	5	2.4-10.2	54.07	0.113
Isospora	1	21.8	12.8-34.6	NA	NA
Giardia lamblia	14	52.1	36.3-67.9	97.08	0
Giardia duodenalis	13	53.4	37.6-68.6	91.25	0
Blastocystis hominis	2	10.5	1.8-42.8	95.5	0
Entamoeba histolytica	10	13.9	6.3-27.8	83.08	0
Endolimax nana	-	-	-	-	-
Entamoeba hartmanni	-	-	-	-	-
Chilomastix mesnili	-	-	-	-	-
Trichomonas hominis	-	-	-	-	-
Cryptosporidium parvum	19	46.2	33.6-59.3	89.74	0

Subgroup Prevalence of Protozoa in America

This meta-analysis summarizes protozoan prevalence in diarrheal cases across the Americas. *Giardia duodenalis* showed the highest pooled prevalence at 56.8% (95% CI: 46.1%-66.9%; I² = 56.63%, p = 0.018), followed closely by *Giardia lamblia* at 50.3% (95% CI: 19.5%-81.0%; I² = 99.04%, p < 0.001), both highlighting significant contributions to protozoan infections. *Cryptosporidium parvum* was identified in 13 studies with a pooled prevalence of 29.9% (95% CI: 17.4%-46.3%; I² = 86.38%). *Entamoeba histolytica* had a prevalence of 25.1% (95% CI: 13.2%-42.4%; I² = 88.11%). Other pathogens with moderate prevalence included *Blastocystis hominis* (32.4%, 95% CI: 25.4%-40.2%) and *Cyclospora cayetanensis* (5.0%, 95% CI: 2.4%-16.0%). Rare protozoa such as *Entamoeba hartmanni*, *Endolimax nana*, *Chilomastix mesnili*, and *Trichomonas hominis* showed consistently low prevalence (2.0-2.6%) with no heterogeneity (I² = 0%). Variability in heterogeneity indicates differences in diagnostic practices and regional burden (Table 4).

**Table 4:** Pooled data on the prevalence of protozoan isolates in the Americas.

Name of enteric pathogen	No. of studies	Prevalence (%)	95% CI	Heterogeneity test (I²)	p-value
Cyclospora cayetanensis	2	5	2.4-16.0	0	0.905
Isospora	1	3.9	1.0-14.3	0	0.463
Giardia lamblia	14	50.3	19.5-81.0	99.04	0
Giardia duodenalis	9	56.8	46.1-66.9	56.63	0.018
Blastocystis hominis	3	32.4	25.4-40.2	23.88	0.269
Entamoeba histolytica	5	25.1	13.2-42.4	88.11	0
Endolimax nana	2	2.6	1.2-5.7	0	0.948
Entamoeba hartmanni	2	2	0.7-5.1	0	0.361
Chilomastix mesnili	2	2	0.7-5.3	1.3	0.314
Trichomonas hominis	2	2	0.7-5.3	1.3	0.314
Cryptosporidium parvum	13	29.9	17.4-46.3	86.38	0

Subgroup Prevalence of Protozoa in Europe

In Europe, *Giardia lamblia* was the most prevalent protozoan pathogen, reported in 9 studies with a pooled prevalence of 54.5% (95% CI: 41.2%-67.8%; I² = 78.71%, p < 0.001). *Giardia duodenalis* followed closely at 50.4% (95% CI: 39.5%-61.3%) across 11 studies, with low heterogeneity (I² = 17.85%, p = 0.274), indicating consistency across studies. *Cryptosporidium parvum* was also common (40.3%; 95% CI: 17.4%-68.3%; I² = 94.28%). *Entamoeba histolytica* had a moderate prevalence of 15.7% (95% CI: 6.2%-34.4%) with high heterogeneity (I² = 80.10%). *Blastocystis hominis* was less frequently studied but showed a high pooled prevalence of 52.6% (95% CI: 12.7%-89.4%; I² = 84.57%). Rare protozoa such as *Chilomastix mesnili* and *Trichomonas hominis* were each reported in a single study, both with 50.0% prevalence (95% CI: 5.9%-94.1%). No data were available for *Cyclospora*, *Isospora*, *Endolimax nana*, and *Entamoeba hartmanni* (Table 5).

**Table 5:** Pooled data on the prevalence of protozoan isolates in Europe.

Name of enteric pathogen	No. of studies	Prevalence (%)	95% CI	Heterogeneity test (I²)	p-value
Cyclospora cayetanensis	-	-	-	-	-
Isospora	-	-	-	-	-
Giardia lamblia	9	54.5	41.2-67.8	78.71	0
Giardia duodenalis	11	50.4	39.5-61.3	17.85	0.274
Blastocystis hominis	2	52.6	12.7-89.4	84.57	0.011
Entamoeba histolytica	7	15.7	6.2-34.4	80.1	0
Endolimax nana	-	-	-	-	-
Entamoeba hartmanni	-	-	-	-	-
Chilomastix mesnili	1	50	5.9-94.1	NA	NA
Trichomonas hominis	1	50	5.9-94.1	NA	NA
Cryptosporidium parvum	17	40.3	17.4-68.3	94.28	0

Subgroup Prevalence of Protozoa in Africa

In Africa, *Giardia duodenalis* had the highest pooled prevalence among protozoan pathogens at 76.4% (95% CI: 57.8%-88.4%; I² = 89.35%, p < 0.001), followed by *Giardia lamblia* at 58.8% (95% CI: 37.6%-79.9%; I² = 97.82%). *Cryptosporidium parvum* was also common, with a prevalence of 47.4% (95% CI: 28.4%-67.2%; I² = 92.48%). In contrast, *Entamoeba histolytica* showed a lower pooled prevalence of 4.7% (95% CI: 1.6%-13.3%; I² = 77.24%). Less prevalent species included *Blastocystis hominis* (10.6%; 95% CI: 2.8%-33.0%) and *Entamoeba hartmanni* (14.4%; 95% CI: 2.0%-58.4%). Rare isolates such as *Endolimax nana* (56.5%) and *Trichomonas hominis* (50.0%) were reported in limited studies with wide CIs. No data were available for *Cyclospora*, *Isospora*, or *Chilomastix mesnili* in African studies (Table 6).

**Table 6:** Pooled data on the prevalence of protozoan isolates in Africa.

Name of organism	No. of studies	Prevalence (%)	95% CI	Heterogeneity test (I²)	p-value
Cyclospora cayetanensis	-	-	-	-	-
Isospora	-	-	-	-	-
Giardia lamblia	12	58.8	37.6-79.9	97.82	0
Giardia duodenalis	8	76.4	57.8-88.4	89.35	0
Blastocystis hominis	2	10.6	2.8-33.0	21.24	0
Entamoeba histolytica	7	4.7	1.6-13.3	77.24	0
Endolimax nana	2	56.5	12.3-92.3	26.44	0.244
Entamoeba hartmanni	2	14.4	2.0-58.4	0	0.876
Chilomastix mesnili	-	-	-	-	-
Trichomonas hominis	1	50	5.9-94.1	NA	NA
Cryptosporidium parvum	12	47.4	28.4-67.2	92.48	0

Global Pooled Prevalence of Protozoan Diarrheal Pathogens

This meta-analysis synthesized data from multiple studies to estimate the global prevalence of protozoan pathogens in diarrheal cases. *Giardia duodenalis* had the highest pooled prevalence at 58.6% (95% CI: 50.8%-66.1%; I² = 84.87%, p < 0.001), followed by *Giardia lamblia* at 53.4% (95% CI: 42.5%-64.2%; I² = 98.15%). *Cryptosporidium parvum* was also prevalent, detected in 61 studies with a pooled estimate of 41.3% (95% CI: 33.1%-50.0%; I² = 91.31%). *Entamoeba histolytica* showed a moderate prevalence of 13.1% (95% CI: 8.7%-19.2%). Less common protozoa included *Blastocystis hominis* (21.4%), *Endolimax nana* (11.5%), *Chilomastix mesnili* (5.7%), and *Trichomonas hominis* (8.9%), all with high heterogeneity. *Cyclospora cayetanensis* and *Isospora* were rare, with prevalence rates of 5.6% and 7.7%, respectively (Table 7). High heterogeneity in most estimates reflects study variability across regions and methodologies.

**Table 7:** Total pooled prevalence estimates for global protozoan isolates.

Name of enteric pathogen	No. of studies	Prevalence (%)	95% CI	Heterogeneity test (I²)	p-value
Cyclospora cayetanensis	5	5.6	3.6-8.6	10.25	0.348
Isospora	3	7.7	1.8-27.2	59.76	0.059
Giardia lamblia	49	53.4	42.5-64.2	98.15	0
Giardia duodenalis	41	58.6	50.8-66.1	84.87	0
Blastocystis hominis	9	21.4	8.5-44.3	97.27	0
Entamoeba histolytica	29	13.1	8.7-19.2	87.2	0
Endolimax nana	4	11.5	2.0-45.5	79.43	0.002
Entamoeba hartmanni	4	3.1	1.0-9.4	25.49	0.259
Chilomastix mesnili	3	5.7	1.0-27.1	66.53	0.03
Trichomonas hominis	4	8.9	1.1-44.4	76.93	0.005
Cryptosporidium parvum	61	41.3	33.1-50.0	91.31	0

Subgroup Prevalence of Global Protozoan Detections Using Various Diagnotics Methods

**Figure 5:**
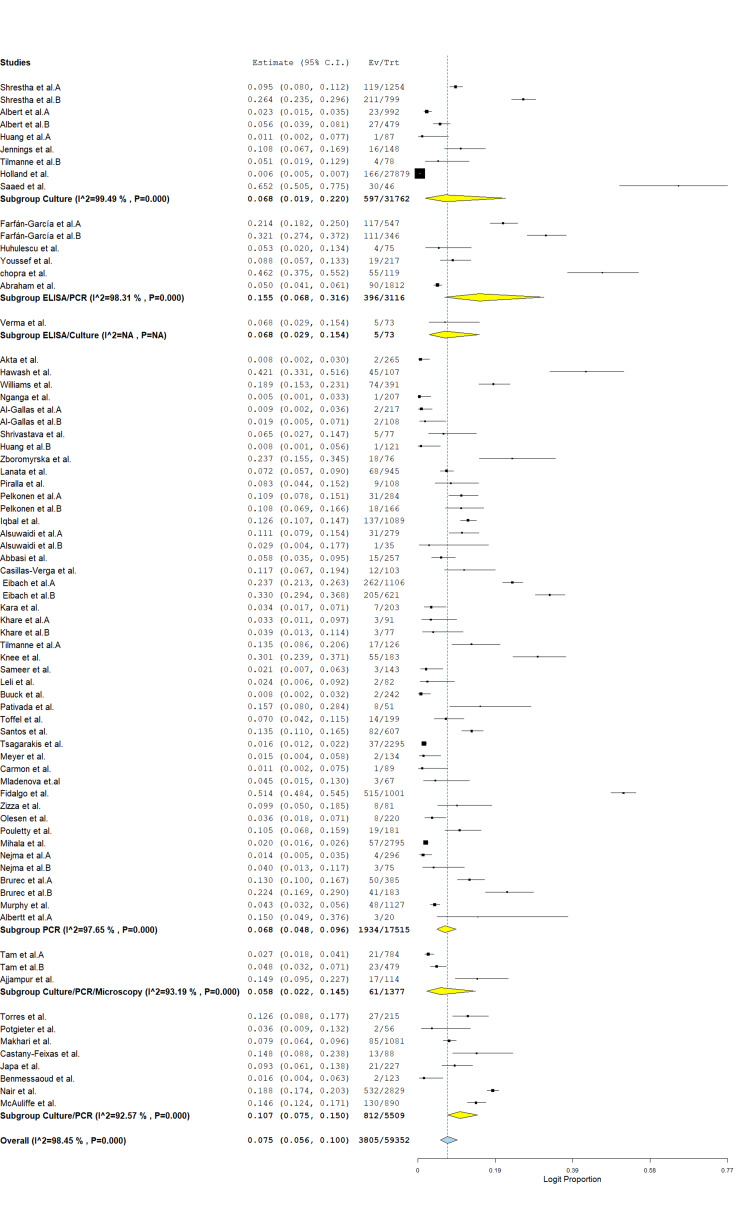
Subgroup prevalence of global protozoan isolates in diarrhea cases based on detection methods. The forest plot was generated using OpenMeta[Analyst] software. Source: References [26-85].

The forest plot presents a comprehensive meta-analysis of multiple studies investigating protozoan detection using various diagnostic methods. The studies are grouped according to the detection technique employed, including PCR, ELISA/Culture, Culture/PCR/Microscopy, Culture/PCR, and Culture alone. Each study is represented with its name, the proportion of positive protozoan detection (referred to as the estimate), the 95% CI, and the number of positive cases over the total examined (Figure 5).

Among the subgroups, studies using PCR exhibited the highest pooled proportion of protozoan detection at 60.9% (95% CI: 49.2%-72.6%), though heterogeneity was extremely high (I² = 99.9%), suggesting substantial variability between studies. The ELISA/Culture subgroup showed a lower pooled detection rate of 50.2% (95% CI: 17.1%-79.2%), again with significant heterogeneity (I² = 99.9%). Similarly, pooled detection rates for the Culture/PCR/Microscopy and Culture/PCR subgroups were 51.4% and 56.7%, respectively. The Culture-only subgroup yielded a pooled detection rate of 60.9% (95% CI: 29.6%-90.4%).

The overall analysis, which combined all included studies regardless of detection method, resulted in a pooled protozoan detection proportion of 59.2% (95% CI: 48.6%-69.8%). However, overall heterogeneity was very high (I² = 99.98%), indicating considerable differences across studies that may stem from varying sample sizes, populations, methodologies, and laboratory techniques.

**Figure 6:**
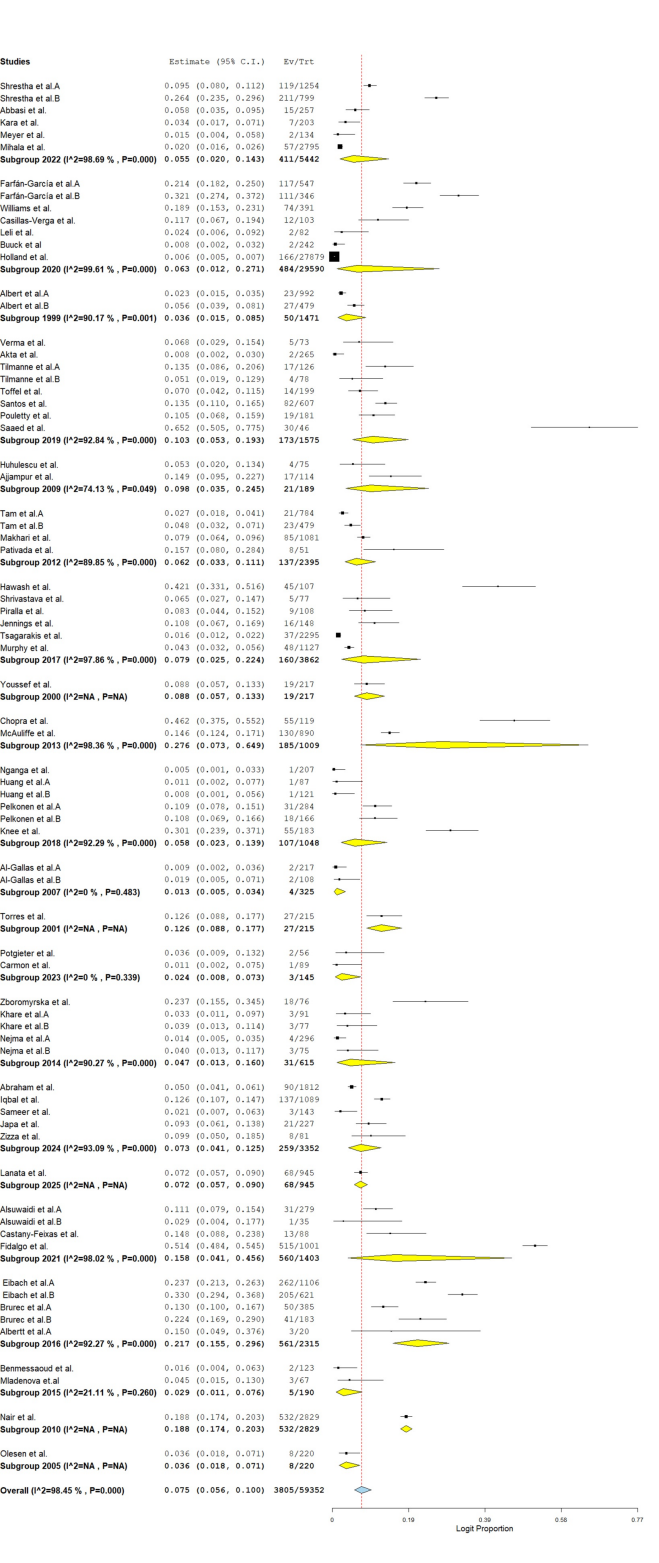
Subgroup prevalence of global protozoan isolates in diarrhea cases based on year of publication. The forest plot was generated using OpenMeta[Analyst] software. Source: References [26-85].

Similarly, the forest plot displays a meta-analysis of protozoan prevalence across multiple studies, grouped by year. Each study’s logit proportion and CI are shown, with subgroup and overall pooled estimates highlighted. The overall prevalence estimate was 0.613 (95% CI: 0.543-0.680), with significant heterogeneity (I² = 99.43%). Most subgroups also demonstrated high heterogeneity (I² > 90%), suggesting variability in protozoan prevalence across studies and years (Figure 6).

Meta-Regression Analysis

**Figure 7:**
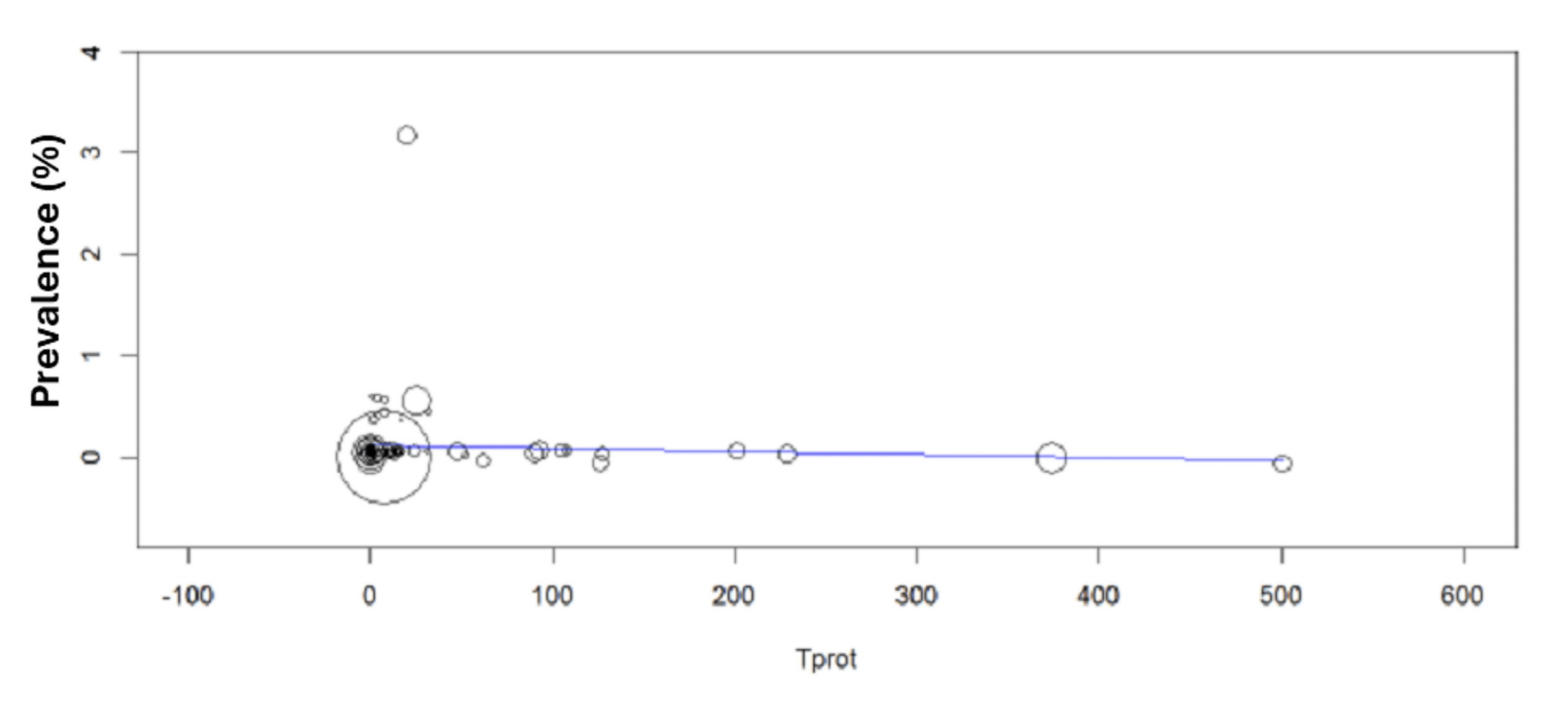
Prevalence of total protozoa vs. total pathogens. Meta-regression analysis was performed using OpenMEE software. Tprot = total protozoa.

**Figure 8:**
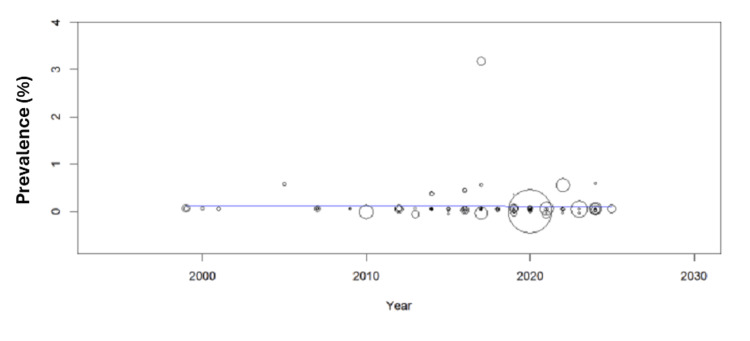
Prevalence of protozoa vs. year of publication. Meta-regression analysis was performed using OpenMEE software.

**Figure 9:**
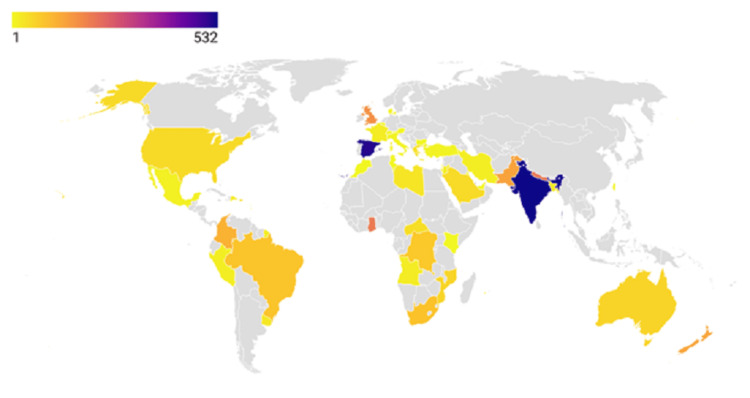
Map showing the distribution of global protozoan isolates across continents. The map was generated using MapChart software.

The meta-regression analyses examining protein-related factors (Tprot) and temporal trends (Year) demonstrated consistent null findings with persistent heterogeneity. For the Tprot analysis, results showed no significant association with effect sizes (β = -0.000, p = 0.533), while revealing substantial residual heterogeneity (I² = 98.62%) that remained unexplained (R² = 0%). The significant intercept (0.116, p = 0.002) indicated baseline effects independent of protein factors. Similarly, the Year analysis found no temporal relationship (β = -0.001, p = 0.868), with nearly identical heterogeneity levels (I² = 98.64%) and no explained variance. The non-significant intercept (p = 0.861) further confirmed the absence of meaningful temporal patterns. Below is a map showing the distribution of global protozoan isolates across continents (Figures 7-9).

Discussion

The global prevalence of enteric protozoa varies considerably across regions, largely influenced by disparities in sanitation, water quality, and socioeconomic conditions [86,87]. Protozoan pathogens such as *Cyclospora cayetanensis*, *Isospora*, and *Giardia duodenalis* pose significant public health risks, particularly in low- and middle-income countries [87].

A recent systematic review estimated that *Cyclospora cayetanensis* is more common in Africa than in other regions, especially in low-income settings [88]. In South America, its prevalence is elevated among individuals living with HIV/AIDS, while the lowest levels are observed in Asia [89]. Environmental factors such as seasonal rainfall and temperature influence transmission, with considerable levels of water contamination reported in endemic areas [90]. Although *Cyclospora cayetanensis* typically causes localized outbreaks, it remains a notable public health concern in Latin America and among children in South Asia [91]. Transmission through contaminated produce and seasonal variability underscores the urgent need for improved food safety in endemic regions. Coincidentally, *Entamoeba histolytica* also remains widespread in South Asia and sub-Saharan Africa [92]. However, our systematic review and meta-analysis found comparable *Cyclospora cayetanensis* prevalence in both Asia and the Americas.

Further, *Giardia duodenalis* continues to be one of the most widespread protozoan pathogens globally, accounting for a substantial number of symptomatic cases each year [93]. Its burden is greatest among children in developing regions, where prevalence rates are significantly higher than in high-income countries [94]. For instance, a Brazilian study identified notable levels of Giardia infection, frequently co-occurring with *Blastocystis hominis* [95,96]. *Entamoeba histolytica* also contributes substantially to gastrointestinal morbidity, particularly among travelers and individuals in endemic areas [97]. The present meta-analysis found that *Giardia duodenalis* is prevalent in Asia, the Americas, Africa, and Europe, while *Entamoeba histolytica* was also commonly detected in these regions, though with variable distribution.

Other protozoan pathogens, including *Blastocystis hominis* and *Cryptosporidium parvum*, exhibit distinct regional patterns. For instance, in sub-Saharan Africa and South Asia, *Blastocystis hominis* is frequently found among children [98-100]. *Cryptosporidium parvum* also demonstrates concerning trends, especially among African children and individuals with compromised immune systems. Furthermore, recent studies have highlighted the prevalence and molecular identification of *Entamoeba histolytica*, Giardia intestinalis, and *Cryptosporidium parvum* in pediatric gastroenteritis, as well as advances in the detection and epidemiology of *Cyclospora cayetanensis*, underscoring their persistent public health importance [101-103]. *Blastocystis hominis* further demonstrates widespread colonization, especially in Southeast Asia and the Middle East [104,105]. Although its pathogenicity remains under debate, growing associations with irritable bowel syndrome and chronic gastrointestinal symptoms warrant additional investigation. These trends highlight the resilience of protozoa in impoverished environments and their disproportionate impact on vulnerable populations. Our study indicated that *Blastocystis hominis* is widespread across all continents, with the highest rates observed in Europe, while *Cryptosporidium parvum* was similarly distributed, with particularly high occurrence in Asia and Africa.

Less frequently studied protozoa such as *Endolimax nana*, *Entamoeba hartmanni*, and *Chilomastix mesnili* have been identified in Africa and South Asia, often as indicators of fecal contamination and substandard hygiene [106]. Though generally nonpathogenic, their presence signals environmental exposure to unsanitary conditions. For example, *Endolimax nana*, which closely resembles *Entamoeba histolytica* morphologically, can be misidentified and is transmitted through the fecal-oral route. Asymptomatic carriers can contribute significantly to environmental contamination, thereby sustaining transmission cycles in affected communities [106-109]. Our review found that *Endolimax nana* and *Entamoeba hartmanni* are more common in Africa than in the Americas, while *Chilomastix mesnili* showed a particularly high occurrence in African settings.

Supporting these findings, our meta-analysis of 73 studies involving over 59,000 diarrheal cases revealed that protozoan infections are globally widespread, with the highest burden reported in the Americas and Africa. *Giardia duodenalis*, *Giardia lamblia*, and *Cryptosporidium parvum* were consistently the most frequently identified species across regions. Although considerable variability existed among studies (I² > 90%), the findings were statistically robust, with minimal publication bias. Diagnostic methods played a critical role in detection, with molecular approaches such as PCR yielding higher prevalence estimates. Meta-regression analysis found no significant link with time trends or protein-related variables, reinforcing the persistent nature of protozoan infections. These results underscore the pressing need for integrated public health strategies that enhance diagnostics, establish routine surveillance, and prioritize sustained investment in water, sanitation, and hygiene (WASH) infrastructure to effectively combat the enduring burden of enteric protozoa globally.

This study’s limitations include high heterogeneity, diagnostic variability, and data gaps from low-resource regions. Most data were cross-sectional and lacked standardized protocols. Future research should employ molecular diagnostics, conduct longitudinal studies, and explore asymptomatic transmission and co-infections. Improved surveillance and WASH interventions are essential to reduce the global burden of enteric protozoan infections.

## Conclusions

This review highlights the significant global burden of enteric protozoan infections, particularly in low- and middle-income regions. The findings underscore the urgent need for improved diagnostics, routine surveillance, and strengthened WASH infrastructure. Addressing these challenges through integrated public health strategies is essential to reduce transmission, improve health outcomes, and protect vulnerable populations from protozoa-related gastrointestinal illnesses worldwide.

## Appendices

Appendix 1

The quality of the 73 included studies was assessed based on the following criteria: (1) appropriate sampling frame to address the target population, (2) appropriate method of sampling study participants, (3) adequate sample size, (4) detailed description of study participants and settings, (5) data analysis with sufficient coverage of the identified sample, (6) use of valid methods to identify the condition, (7) standard and reliable measurement of the condition for all participants, (8) availability of appropriate statistical analysis, and (9) adequate response rate with proper management of low response rates. The quality of the included studies was evaluated using the JBI critical appraisal checklist for studies reporting prevalence data.

**Table 8:** Quality of included studies by JBI critical appraisal checklist for studies reporting prevalence data References [26-85]

Study name		Checklist*									Overall
		1	2	3	4	5	6	7	8	9	
1	Shrestha et al.A [26]	Yes	No	Yes	Yes	Yes	Yes	Yes	Yes	Yes	7
2	Shrestha et al.B [26]	Yes	No	Yes	No	Yes	Yes	Yes	Yes	Yes	7
3	Farfán-García et al.A [27]	Yes	No	Yes	Yes	Yes	Yes	Yes	Yes	Yes	8
4	Farfán-García et al.B [27]	Yes	No	Yes	Yes	Yes	Yes	Yes	Yes	Yes	8
5	Albert et al.A [28]	Yes	Yes	Yes	Yes	Yes	Yes	Yes	Yes	Yes	9
6	Albert et al.B [28]	Yes	Yes	Yes	Yes	Yes	Yes	Yes	Yes	Yes	9
7	Verma et al. [29]	Yes	Yes	Yes	No	Yes	Yes	Yes	Yes	Yes	8
8	Huhulescu et al. [30]	Yes	No	Yes	No	Yes	Yes	Yes	Yes	Yes	7
9	Aktaş et al. [31]	Yes	No	Yes	No	Yes	Yes	Yes	Yes	Yes	8
10	Tam et al.A [32]	Yes	No	Yes	Yes	Yes	Yes	Yes	Yes	Yes	8
11	Tam et al.B [32]	Yes	No	Yes	Yes	Yes	Yes	Yes	Yes	Yes	8
12	Hawash et al. [33]	Yes	No	Yes	No	Yes	Yes	Yes	Yes	Yes	7
13	Youssef et al. [34]	Yes	No	Yes	No	Yes	Yes	Yes	Yes	Yes	7
14	Williams et al. [35]	Yes	No	Yes	No	Yes	Yes	Yes	Yes	Yes	7
15	chopra et al. [36]	Yes	No	Yes	Yes	Yes	Yes	Yes	Yes	Yes	8
16	Ng’ang’a et al. [37]	Yes	No	Yes	Yes	Yes	Yes	Yes	Yes	Yes	8
17	Ajjampur et al. [38]	Yes	No	Yes	Yes	Yes	Yes	Yes	Yes	Yes	8
18	Al-Gallas et al.A [39]	Yes	No	Yes	NO	Yes	Yes	Yes	Yes	Yes	7
19	Al-Gallas et al.B [39]	Yes	No	Yes	Yes	Yes	Yes	Yes	Yes	Yes	7
20	Torres et al. [40]	Yes	No	Yes	Yes	Yes	Yes	Yes	Yes	Yes	8
21	Potgieter et al. [41]	Yes	No	Yes	No	Yes	Yes	Yes	Yes	Yes	7
22	Makhari et al. [42]	Yes	No	Yes	Yes	Yes	Yes	Yes	Yes	Yes	8
23	Shrivastava et al. [43]	Yes	Yes	Yes	Yes	Yes	Yes	Yes	Yes	Yes	9
24	Huang et al.A [44]	Yes	No	Yes	Yes	Yes	Yes	Yes	Yes	Yes	8
25	Huang et al.B [44]	Yes	No	Yes	Yes	Yes	Yes	Yes	Yes	Yes	8
26	Zboromyrska et al. [45]	Yes	No	Yes	Yes	Yes	Yes	Yes	Yes	Yes	8
27	Abraham et al. [46]	Yes	No	Yes	Yes	Yes	Yes	Yes	Yes	Yes	9
28	Lanata et al. [47]	Yes	No	Yes	Yes	Yes	Yes	Yes	Yes	Yes	8
29	Piralla et al. [48]	Yes	No	Yes	Yes	Yes	Yes	Yes	Yes	Yes	8
30	Pelkonen et al.A [49]	Yes	No	Yes	Yes	Yes	Yes	Yes	Yes	Yes	8
31	Pelkonen et al.B [49]	Yes	No	Yes	No	Yes	Yes	Yes	Yes	Yes	7
32	Iqbal et al. [50]	Yes	No	Yes	Yes	Yes	Yes	Yes	Yes	Yes	8
33	Alsuwaidi et al.A [51]	Yes	No	Yes	Yes	Yes	Yes	Yes	Yes	Yes	8
34	Alsuwaidi et al.B [51]	Yes	No	Yes	Yes	Yes	Yes	Yes	Yes	Yes	8
35	Abbasi et al. [52]	Yes	No	Yes	No	Yes	Yes	Yes	Yes	Yes	7
36	Casillas-Verga et al. [53]	Yes	No	Yes	Yes	Yes	Yes	Yes	Yes	Yes	8
37	Jennings et al. [54]	Yes	No	Yes	Yes	Yes	Yes	Yes	Yes	Yes	8
38	Eibach et al.A [55]	Yes	No	Yes	Yes	Yes	Yes	Yes	Yes	Yes	8
39	Eibach et al.B [55]	Yes	No	Yes	Yes	Yes	Yes	Yes	Yes	Yes	8
40	Kara et al. [56]	Yes	No	Yes	Yes	Yes	Yes	Yes	Yes	Yes	8
41	Khare et al.A [57]	Yes	No	Yes	Yes	Yes	Yes	Yes	Yes	Yes	8
42	Khare et al.B [57]	Yes	No	Yes	Yes	Yes	Yes	Yes	Yes	Yes	8
43	Tilmanne et al.A [58]	Yes	No	Yes	Yes	Yes	Yes	Yes	Yes	Yes	8
44	Tilmanne et al.B [58]	Yes	No	Yes	Yes	Yes	Yes	Yes	Yes	Yes	8
45	Castany-Feixas et al. [59]	Yes	No	Yes	Yes	Yes	Yes	Yes	Yes	Yes	8
46	Knee et al. [60]	Yes	No	Yes	Yes	Yes	Yes	Yes	Yes	Yes	8
47	Sameer et al. [61]	Yes	No	Yes	Yes	Yes	Yes	Yes	Yes	Yes	8
48	Japa et al. [62]	Yes	No	Yes	Yes	Yes	Yes	Yes	Yes	Yes	8
49	Benmessaoud et al. [63]	Yes	No	Yes	Yes	Yes	Yes	Yes	Yes	Yes	8
50	Leli et al. [64]	Yes	No	Yes	Yes	Yes	Yes	Yes	Yes	Yes	8
51	Buuck et al. [65]	Yes	No	Yes	Yes	Yes	Yes	Yes	Yes	Yes	8
52	Pativada et al. [66]	Yes	No	Yes	No	Yes	Yes	Yes	Yes	Yes	7
53	Toffel et al. [67]	Yes	No	Yes	Yes	Yes	Yes	Yes	Yes	Yes	8
54	Nair et al. [68]	Yes	No	Yes	No	Yes	Yes	Yes	Yes	Yes	7
55	Santos et al. [69]	Yes	No	Yes	Yes	Yes	Yes	Yes	Yes	Yes	8
56	Tsagarakis et al. [70]	Yes	No	Yes	Yes	Yes	Yes	Yes	Yes	Yes	8
57	Meyer et al. [71]	Yes	No	Yes	Yes	Yes	Yes	Yes	Yes	Yes	7
58	Carmon et al. [72]	Yes	No	Yes	Yes	Yes	Yes	Yes	Yes	Yes	8
59	Mladenova et.al [73]	Yes	Yes	Yes	Yes	Yes	Yes	Yes	Yes	Yes	9
60	Fidalgo et al. [74]	Yes	No	Yes	Yes	Yes	Yes	Yes	Yes	Yes	8
61	McAuliffe et al. [75]	Yes	Yes	Yes	No	Yes	Yes	Yes	Yes	Yes	8
62	Holland et al. [76]	Yes	No	Yes	No	Yes	Yes	Yes	Yes	Yes	7
63	Zizza et al. [77]	Yes	No	Yes	No	Yes	Yes	Yes	Yes	Yes	8
64	Olesen et al. [78]	Yes	No	Yes	Yes	Yes	Yes	Yes	Yes	Yes	8
65	Pouletty et al. [79]	Yes	No	Yes	Yes	Yes	Yes	Yes	Yes	Yes	8
66	Mihala et al. [80]	Yes	No	Yes	No	Yes	Yes	Yes	Yes	Yes	7
67	Nejma et al.A [81]	Yes	No	Yes	No	Yes	Yes	Yes	Yes	Yes	7
68	Nejma et al.B [81]	Yes	No	Yes	No	Yes	Yes	Yes	Yes	Yes	7
69	Saaed et al. [82]	Yes	No	Yes	Yes	Yes	Yes	Yes	Yes	Yes	8
70	Brurec et al.A [83]	Yes	No	Yes	Yes	Yes	Yes	Yes	Yes	Yes	8
71	Brurec et al.B [83]	Yes	No	Yes	Yes	Yes	Yes	Yes	Yes	Yes	8
72	Murphy et al. [84]	Yes	No	Yes	NO	Yes	Yes	Yes	Yes	Yes	7
73	Albertt et al.A [85]	Yes	No	Yes	Yes	Yes	Yes	Yes	Yes	Yes	7

Appendix 2

Search Strategy Word (Dec 30, 2024). Total Search = 1133

Web of Science (N = 164) (Dec 30, 2024)

https://www.webofscience.com/wos/alldb/summary/9cc2ac32-4cb4-423e-8197-6e88b24ca0c4-0144be2588/relevance/1

(((((((((((((((((((((((((((((((((((ALL=(Coinfection)) OR ALL=(Concurrent infection)) OR ALL=(Dual infection)) AND ALL=(Prevalence)) OR ALL=(Incidence)) OR ALL=(Occurrence)) AND ALL=(Cholera)) OR ALL=(Vibrio cholerae)) OR ALL=(V. cholerae)) OR ALL=(Vibrio cholerae infection)) OR ALL=(Cholera disease)) OR ALL=(Cholera outbreaks)) OR ALL=(Cholera epidemic)) AND ALL=(Shigella)) OR ALL=(Shigellosis)) OR ALL=(Shigella-induced diarrhea)) OR ALL=(Shigella enteric infection)) AND ALL=(Escherichia coli)) OR ALL=(E. coli infection)) OR ALL=(Pathogenic E. coli)) OR ALL=(Enteropathogenic E. coli)) AND ALL=(Campylobacter)) OR ALL=(Campylobacter infection)) OR ALL=(Campylobacteriosis)) OR ALL=(Campylobacter enteric infection)) AND ALL=(Rotavirus)) OR ALL=(Rotavirus infection)) OR ALL=(Rotaviral gastroenteritis)) OR ALL=(Rotavirus-induced diarrhea)) AND ALL=(Norovirus)) OR ALL=(Norovirus infection)) OR ALL=(Noroviral gastroenteritis)) OR ALL=(Norovirus-induced diarrhea)) AND ALL=(Cryptosporidium)) OR ALL=(Cryptosporidiosis)) AND ALL=(Salmonella)

PubMed (N = 9) (Dec 30, 2024) (Coinfection[Title/Abstract]) OR (Co-infection[Title/Abstract]) AND (Prevalence[Title/Abstract]) OR (Incidence[Title/Abstract]) AND (Cholera[Title/Abstract]) OR (Vibrio cholera[Title/Abstract]) OR (V. cholera[Title/Abstract]) AND (Shigella[Title/Abstract]) OR (Shigellosis[Title/Abstract]) AND (Escherichia coli[Title/Abstract]) OR (E. coli[Title/Abstract]) AND (Campylobacter[Title/Abstract]) AND (Rotavirus[Title/Abstract]) AND (Norovirus[Title/Abstract]) AND (Cryptosporidium[Title/Abstract])

PubMed (N = 17) (Dec 30, 2024) - MeSH Combination "Coinfection"[Mesh] OR "Coinfection/microbiology"[Mesh] OR "Coinfection/parasitology"[Mesh] OR "Coinfection/prevention and control"[Mesh] OR "Coinfection/virology"[Mesh] OR "Prevalence"[Mesh] AND "Epidemiology"[Mesh] AND "epidemiology"[Subheading] OR "Incidence"[Mesh] AND "Epidemiology"[Mesh] AND "epidemiology"[Subheading] AND "Cohort Studies"[Mesh] OR "Cholera"[Mesh] AND "Vibrio cholerae"[Mesh] AND "Cholera Vaccines"[Mesh] OR "Shigella"[Mesh] AND "Shigella dysenteriae"[Mesh] AND "Shigella Vaccines"[Mesh] OR ("Dysentery, Bacillary/microbiology"[Mesh] OR "Dysentery, Bacillary/parasitology"[Mesh] OR "Dysentery, Bacillary/prevention and control"[Mesh] OR "Dysentery, Bacillary/virology"[Mesh]) OR "Escherichia coli"[Mesh] AND "Escherichia coli Vaccines"[Mesh] OR "Rotavirus"[Mesh] AND "Rotavirus Vaccines"[Mesh] AND "Rotavirus Infections"[Mesh] OR "Campylobacter"[Mesh] AND "Campylobacter Infections"[Mesh] OR "Norovirus"[Mesh] AND "Caliciviridae Infections"[Mesh] AND "Salmonella"[Mesh]

Scopus Format (N = 39) (Dec 30, 2024) TITLE-ABS ((coinfection OR "Co-infection" OR "Concurrent infection" OR "Dual infection" ) AND ( prevalence OR incidence OR occurrence ) AND ( cholera OR "Vibrio cholerae" OR "V. cholerae" OR "Vibrio cholerae infection" OR "Cholera disease" OR "Cholera outbreaks" OR "Cholera epidemic" ) AND ( shigella OR shigellosis OR "Shigella-induced diarrhea" OR "Shigella enteric infection" ) AND ( "Escherichia coli" OR "E. coli infection" OR "Pathogenic E. coli" OR "Enteropathogenic E. coli" ) AND ( campylobacter OR "Campylobacter infection" OR campylobacteriosis OR "Campylobacter enteric infection" ) AND ( rotavirus OR "Rotavirus infection" OR "Rotaviral gastroenteritis" OR "Rotavirus-induced diarrhea" ) AND ( norovirus OR "Norovirus infection" OR "Noroviral gastroenteritis" OR "Norovirus-induced diarrhea" ) AND ( cryptosporidium OR "Cryptosporidium infection" OR cryptosporidiosis ) AND salmonella)

Google Scholar (N = 785) (Dec 30, 2024) allintitle: ( ( coinfection OR "Co-infection" OR "Concurrent infection" OR "Dual infection" ) AND ( prevalence OR incidence OR occurrence ) AND ( cholera OR "Vibrio cholerae" OR "V. cholerae" OR "Vibrio cholerae infection" OR "Cholera disease" OR "Cholera outbreaks" OR "Cholera epidemic" ) AND ( shigella OR shigellosis OR "Shigella-induced diarrhea" OR "Shigella enteric infection" ) AND ( "Escherichia coli" OR "E. coli infection" OR "Pathogenic E. coli" OR "Enteropathogenic E. coli" ) AND ( campylobacter OR "Campylobacter infection" OR campylobacteriosis OR "Campylobacter enteric infection" ) AND ( rotavirus OR "Rotavirus infection" OR "Rotaviral gastroenteritis" OR "Rotavirus-induced diarrhea" ) AND ( norovirus OR "Norovirus infection" OR "Noroviral gastroenteritis" OR "Norovirus-induced diarrhea" ) AND ( cryptosporidium OR "Cryptosporidium infection" OR cryptosporidiosis ) AND salmonella )

Science Direct (N = 128) (Dec 30, 2024) - Search by Title, Abstract, or Author-Specified Keywords ("Coinfection" OR "Co-infection") ("Prevalence" OR "Incidence") ("Cholera" OR "Vibrio cholera" OR "V. cholera") ("Shigella" OR "Shigellosis") ("Escherichia coli" OR "E. coli infection") ("Campylobacter") ("Rotavirus") ("Norovirus") ("Cryptosporidium") ("Salmonella")

Appendix 3

**Table 9:** Distribution of various protozoan species in diarrhea cases from 1999-2024. Source: References [26-85].

S/N	Author	Publication Year	Country	Continent	Case sample size	Total pathogens	Total protozoan	Cyclospora cayetanensis	Isospora	Giardia lamblia	Giardia duodenalis	Blastocystis hominis	Entamoeba histolytica	Endolimax nana	Entamoeba hartmanni	Chilomastix mesnili	Trichomonas hominis	Morganella morganii	Cryptosporidium parvum
1	Shrestha et al. A [26]	2022	Nepal	Asia	1200	1254	119	10	0	83	0	0	0	0	0	0	0	0	26
2	Shrestha et al. B [26]	2022	Nepal	Asia	1200	799	211	8	0	193	0	0	0	0	0	0	0	0	10
3	Farfán-García et al. A [27]	2020	Colombia	America	431	547	117	0	0	0	24	43	31	3	3	1	0	0	12
4	Farfán-García et al. B [27]	2020	Colombia	America	430	346	111	0	0	0	25	33	43	3	1	3	0	0	3
5	Albert et al. A [28]	1999	Bangladesh	Asia	814	992	23	0	0	7	0	0	5	0	0	0	0	0	11
6	Albert et al. B [28]	1999	Bangladesh	Asia	814	479	27	0	0	23	0	0	1	0	0	0	0	0	3
7	Verma et al. [29]	2019	India (North)	Asia	100	73	5	0	0	0	4	0	1	0	0	0	0	0	0
8	Huhulescu et al. [30]	2009	Austria	Europe	306	75	4	0	0	2	0	0	0	0	0	0	0	0	2
9	Aktaş et al. [31]	2019	Turkey	Europe	375	265	2	0	0	0	0	0	0	0	0	1	1	0	0
10	Tam et al. A [32]	2012	England	Europe	874	784	21	0	0	9	0	0	0	0	0	0	0	0	12
11	Tam et al. B [32]	2012	England	Europe	782	479	23	0	0	15	0	0	0	0	0	0	0	0	8
12	Hawash et al. [33]	2017	Saudi Arabia	Asia	163	107	45	0	0	0	27	0	4	0	0	0	0	0	14
13	Youssef et al. [34]	2000	Jordan	Asia	265	217	19	0	0	2	0	0	13	0	0	0	0	0	4
14	Williams et al. [35]	2020	Democratic Republic of the Congo	Africa	269	391	74	0	0	0	0	0	0	0	0	0	0	0	74
15	Chopra et al. [36]	2013	India	Asia	200	119	55	1	12	0	0	0	0	0	0	0	0	0	42
16	Ng’ang’a et al. [37]	2018	Kenya	Africa	174	207	1	0	0	0	0	0	0	0	0	0	0	1	0
17	Ajjampur et al. [38]	2009	India	Asia	452	114	17	0	0	0	0	0	0	0	0	0	0	0	17
18	Al-Gallas et al. A [39]	2007	Tunisia	Africa	271	217	2	0	0	2	0	0	0	0	0	0	0	0	0
19	Al-Gallas et al. B [39]	2007	Tunisia	Africa	271	108	2	0	0	0	0	0	0	2	0	0	0	0	0
20	Torres et al. [40]	2001	Uruguay	America	224	215	27	0	0	8	0	0	0	0	0	0	0	0	19
